# Editorial: Optimising Antibiotic Use: Social and Contextual Issues

**DOI:** 10.3389/fsoc.2020.620048

**Published:** 2021-01-12

**Authors:** Carolyn Tarrant, Eva Maria Krockow, Emily Kate Rousham, Björn Rönnerstrand

**Affiliations:** ^1^Department of Health Sciences, University of Leicester, Leicester, United Kingdom; ^2^Department of Neuroscience, Psychology and Behavior, University of Leicester, Leicester, United Kingdom; ^3^School of Sport, Exercise and Health Sciences, Loughborough University, Loughborough, United Kingdom; ^4^The SOM Institute, University of Gothenburg, Gothenburg, Sweden

**Keywords:** antibiotic prescribing, social theory, behavior, context, antimicrobial resistance

Modern medicine is founded on the availability of effective antimicrobials, but antibiotic resistance is an inevitable consequence of widespread use of antibiotics. The rise in multidrug resistant organisms, and the increasing numbers of pan-resistant infections being identified, mean that antimicrobial resistance (AMR) is now a pressing global concern (Jee et al., 2018).

The factors contributing to the growth and spread of AMR are multiple and intertwined, including antibiotic use in human and animal health and agriculture, hygiene and sanitation, and international travel. Overuse of antibiotics in healthcare is a contributing factor, with evidence suggesting that around 30% of antibiotics in hospitals are prescribed or taken inappropriately (Fridkin et al., 2014). The global COVID-19 pandemic has led to increased antibiotic prescribing and made it more difficult to maintain efforts to optimize antibiotic use (Getahun et al., 2020).

Given the global nature of the threat of AMR, efforts are underway to tackle the problem using approaches such as policy, regulation, and international coordination. Alongside these macro-level approaches, an extensive body of research and interventions focuses on understanding and changing the behaviors of individuals—health professionals and patients—to reduce antibiotic use (Charani et al., 2011). In contrast, meso-level issues, including the social and organizational context in which antibiotic use takes place, have received less attention. Prescribing behavior is socially and contextually embedded—influenced by identities, attitudes, social norms, hierarchy, local culture and systems (Charani et al., 2013; Broom et al., 2016). Antibiotic use involves a range of stakeholders and institutions (Broom et al., 2019). The articles in this Research Topic provide insights into the complex and embedded nature of antibiotic use.

Five of the articles in this Research Topic consider antibiotic prescribing and antimicrobial stewardship (programmes to optimize antibiotic use) in healthcare. The first article in the collection, by Rzewuska et al., sets the scene by reviewing the literature on antimicrobial stewardship interventions in hospitals in high income countries. The authors identify that efforts to change physician prescribing behavior are frustrated by contextual factors—inadequate information systems and unavailability of key personnel and funding—as well as facing competition from other initiatives seen as having a higher priority.

Rynkiewich's article goes further by challenging a common assumption of antibiotic stewardship approaches—that the problem lies in individual physician behaviors and habits. Based on vivid ethnographic case studies in two American hospitals, the author argues that decision-making about antibiotics is instead a collective practice, which happens between institutions, physicians, pharmacists, nurses, and other staff. The implications are that, rather than targeting “bad” physician practice, stewardship efforts should recognize the collective nature of antibiotic use.

Tarrant et al. look in detail at the notion of “inappropriate” antibiotic prescribing in their interview study, involving hospital prescribers in the UK, South Africa and Sri Lanka. They identify that prescriber definitions of inappropriate antibiotic use are not purely objective. Inappropriate antibiotic use can't be pinned down by precise technical definitions. Instead, prescribers' judgements about the appropriateness of prescribing decisions reflect their moral position and the context in which they work.

Shifting the focus to China, Chen et al.'s qualitative study provides another example of the complexities of antibiotic decision-making, this time in rural primary care. They find that physicians work to balance their understanding of rational use of antibiotics against the need to maintain good relationships and protect the safety of their patients. The idea of “suzhi” (human quality)—respecting and protecting their patients—is drawn on by prescribers to explain their liberal prescribing of antibiotics.

How patients and professionals perceive suspected infection is consequential for antibiotic use. Saukko and Rousham's article focuses on patients' and health professionals' affective experiences related to diagnosis and management of urinary tract infections. Using the conceptual framing of illness narratives, they describe narratives of chaos and control, and point to the need for stewardship programmes to consider the affective dimension of decision-making about antibiotic use.

The majority of efforts to tackle AMR to date have focused on the preservation of existing antibiotics through stewardship, and investment in development of new antibiotics. The final article in this Topic, by Jamie and Sharples, offers a refreshingly different perspective. The authors describe how natural materials such as clay may offer alternatives to antibiotic treatment. They provide some theoretical lenses through which sociologists could study how their materiality (i.e., what they are made from, what they look like, how they are produced) might influence the use of these materials in healthcare.

Taken together, the articles illuminate the ways in which the use of antibiotics (and materials with antibacterial properties) are shaped by social and organizational infrastructures, individual meaning-making, as well as the materiality of the substances themselves. Addressing AMR will require us to think differently about the nature of the problem and about our possible futures (Chandler, 2019). This Research Topic offers some new perspectives to stimulate further research.

## Author Contributions

The authors confirm being the only contributors of this work and approved it for publication. All authors contributed to the article and approved the submitted version.

## Conflict of Interest

The authors declare that the research was conducted in the absence of any commercial or financial relationships that could be construed as a potential conflict of interest.
